# Conserved Conformational Dynamics Reveal a Key Dynamic Residue in the Gatekeeper Loop of Human Cyclophilins

**DOI:** 10.1021/acs.jpcb.2c08650

**Published:** 2023-03-29

**Authors:** Furyal Ahmed, Xin-Qiu Yao, Donald Hamelberg

**Affiliations:** † Department of Chemistry, Georgia State University, Atlanta, Georgia 30302-3965, United States; ‡ Agnes Scott College, Decatur, Georgia 30030, United States

## Abstract

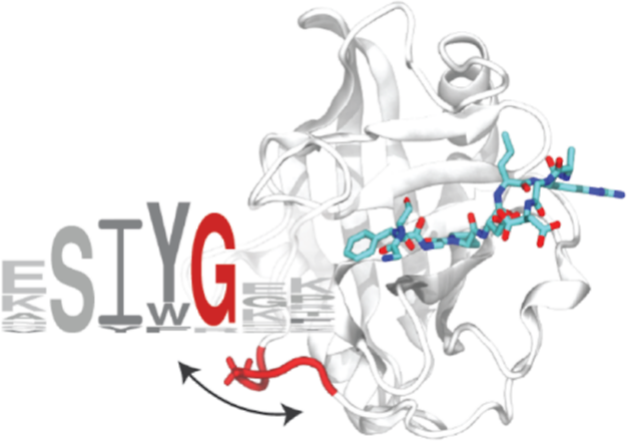

Cyclophilins are ubiquitous human enzymes that catalyze peptidyl-prolyl *cis*–*trans* isomerization in protein substrates. Of the 17 unique isoforms, five closely related isoforms (CypA–E) are found in various environments and participate in diverse cellular processes, yet all have similar structures and the same core catalytic function. The question is what key residues are behind the conserved function of these enzymes. Here, conformational dynamics are compared across these isoforms to detect conserved dynamics essential for the catalytic activity of cyclophilins. A set of key dynamic residues, defined by the most dynamically conserved positions, are identified in the gatekeeper 2 region. The highly conserved glycine (Gly80) in this region is predicted to underlie the local flexibility, which is further tested by molecular dynamics simulations performed on mutants (G80A) of CypE and CypA. The mutation leads to decreased flexibility of CypE and CypA during substrate binding but increased flexibility during catalysis. Dynamical changes occur in the mutated region and a distal loop downstream of the mutation site in sequence. Examinations of the mutational effect on catalysis show that both mutated CypE and CypA exhibit shifted binding free energies of the substrate under distinct isomer conformations. The results suggest a loss of function in the mutated CypE and CypA. These catalytic changes by the mutation are likely independent of the substrate sequence, at least in CypA. Our work presents a method to identify function-related key residues in proteins.

## Introduction

Conformational dynamics have been increasingly recognized to be essential for protein function, including enzyme catalysis, protein–ligand interactions, and allosteric regulation.^1−6^ From a theoretical point of view, a protein molecule is a complex system sampling multiple nearly isoenergetic conformational substates defined in the rugged bottom of the energy landscape.^1^ Perturbation (such as a mutation or small-molecule binding) alters the landscape, causing a population shift of the conformational ensemble toward a modified function. Protein evolution illustrates this fine tuning of protein dynamics and function, where sequence variations (mutations) have produced a spectrum of specific functions observed in a protein family. Hence, evolution provides a unique opportunity for exploring the relationship between dynamics and function. By comparing conformational dynamics of extant homologous proteins^7−11^ or their reconstructed ancestors,^12^ we can identify conserved dynamics that may underlie the core functionality of the family inherited from the common ancestor and divergent dynamics that may be related to functional specificities.

**Figure 1:**
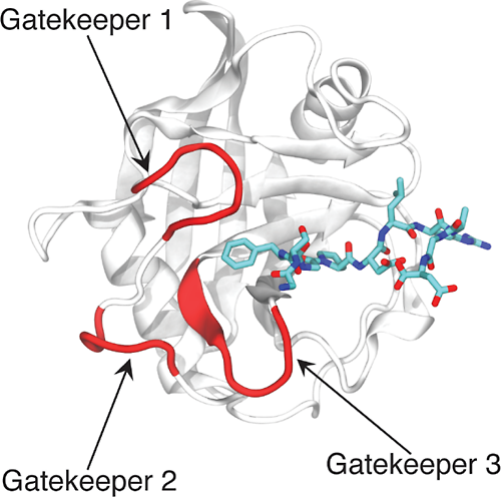
Structure of CypA. The crystallographic structure of CypA (PDB: 3K0M) is shown as a cartoon. The gatekeeper loops are colored red. The modeled peptide substrate (Gly-Pro) is shown as sticks, color coded by atom types.

Cyclophilins are tractable model systems for understanding the role of dynamics in enzyme catalysis.^13,14^ Cyclophilins belong to a class of proteins known as peptidyl-prolyl isomerases (PPIases), which catalyze the interconversion of the peptide bond preceding a proline between the *cis* and *trans* isomers. This reaction is rate-limiting in many biological processes. Cyclophilins have been implicated in a variety of cellular processes, including protein folding, regulation of apoptosis, immunosuppression, and RNA splicing. They have also been reported to be involved in diseases such as human immunodeficiency virus (HIV) and SARS-CoV-2 virus infections and cancer^15−17^ and so are important therapeutic targets. Cyclophilins are found in all organisms discovered so far. All cyclophilin isoforms are structurally conserved, and almost all have PPIase activity.^15^ They are characterized by the presence of a common domain consisting of approximately 109 amino acids, named the cyclophilin-like domain (CLD). The CLD is further surrounded by unique domains associated with the specific function of each isoform.^18^ Another important feature of the cyclophilin structure is the presence of three loop regions, called gatekeeper 1–3, which control catalysis by restricting access to the active site (Figure 1).^15^

Among the 17 human cyclophilins, CypA is the best studied. The overexpression of CypA is linked to the development of several types of cancer.^19,20^ As a potential therapeutic target, CypA interacts with various drugs such as the immunosuppressive drug cyclosporine A and anti-cancer drugs, which have been shown to suppress the rejection of organ transplants and reduce the overexpression of CypA itself, respectively. CypA also regulates the infection by various viruses like HIV-1 and hepatitis C. Several efforts have been made to design and develop potent isoform-specific inhibitors for CypA and other cyclophilin isoforms.^21−24^ Insights into the dynamics of CypA and other cyclophilins and how it can be modified by variations of the primary structure may provide information needed to develop new isoform-specific cyclophilin inhibitors and also to engineer new enzymes for better PPIase activities.

In this study, we focused on five closely related cyclophilin isoforms (CypA–E; Figure S1). Because CypA–E are found in a variety of environments and implicated in diverse cellular pathways, their conserved functional dynamics can provide insight into their conserved function involving the isomerization of the proline peptide bond. We have previously characterized and compared the conformational dynamics of these isoforms using a combined approach of molecular dynamics (MD) simulations and primarily residue–residue contact analysis.^11,25^ In this work, we first re-analyzed those simulation data using several newly developed statistical methods including the recent dihedral principal component analysis (dPCA+)^26^ and the new “mixed-ensemble” Cartesian-based and angular variance analyses. A glycine (G80; CypA numbering) was identified to be a key residue for the functional dynamics of cyclophilins. New simulations were then performed and analyzed to evaluate the mutational effect of G80A on the conformational dynamics and catalytic activity in the contexts of distinct cyclophilins and peptide substrates.

## Methods

### Crystallographic Structure Preparation

Crystallographic structures were obtained from the RCSB Protein Data Bank (PDB),^27^ with entry IDs 3K0M, 3ICH, 2ESL, 4O8H, and 3UCH for CypA–E, respectively. Protein sequences were aligned with MUSCLE^28^ using the Bio3D^29−31^ package to find equivalent residues across isoforms. Subsequent analyses used the alignment numbering; additional residues which were not aligned with all isoforms were excluded from the root-mean-square fluctuation (RMSF) and angular variance analyses.

### Molecular Dynamics Simulations

Wildtype (WT) MD simulations for CypA–E with the Gly-Ser-Phe-**Gly-Pro**-Asp-Leu-Arg-Ala-Gly-Asp (Gly-Pro) peptide were performed as previously described.^11,25^ MD simulations for the mutant isoforms as well as CypA with the Gly-Ser-Phe-**Ala-Pro**-Asp-Leu-Arg-Ala-Gly-Asp (Ala-Pro) (Ala-Pro) peptide were performed using AMBER16^32^ along with the AMBER ff14SB force field.^33^ Reoptimized parameters for the backbone ω-torsion angle were also used, which have been shown to better describe the energetics of prolyl isomerization than the original AMBER force field.^34^ The protonation state of ionizable residues was determined by the H++ web server^35^ at pH 7.0, a salt concentration of 0.15 M, an external dielectric of 80, and an internal dielectric of 10. The tautomer states of neutral histidine residues (HID or HIE) were further examined by visual inspections of the local structural environment surrounding histidine. Both CypA and CypE G80A mutants were constructed by changing the residue name to ALA in the structures. Missing atoms of alanine were then added in by AMBER16. The initial coordinates for the substrate-free and *cis* states were taken from a previously performed simulation of the appropriate isoform. The initial coordinates for the ts substrate were generated via a sequence of restrained MD as previously described until ω_0_ = 90°.^11^ The coordinates for the *trans* substrate were generated via a similar method; the final frame from the restrained MD of the transition state was used as the starting point, and restrained MD was performed until ω_0_ = 180°. The Ala-Pro peptide was generated by modifying the peptide PDB file to the appropriate residues centered at the catalytic proline by keeping the common atoms and using AMBER16 to add the missing atoms.

Each system was solvated and equilibrated as previously described.^11^ Production MD was then performed for 2.1 to 2.7 μs under the same conditions as equilibration. Only the last 2 μs of simulation data at equilibrium, as determined by the root mean square deviation, were used for analysis in all systems. Constant temperature (300 K; Langevin thermostat with collision frequency γ = 1.0 ps^–1^) and constant pressure (1 bar; Monte Carlo barostat with a coupling constant τ_p_ = 1.0 ps) along with periodic boundary conditions were applied. The particle-mesh Ewald summation method^36^ was employed to treat the long-range electrostatic interactions. For short-range nonbonded interactions, a 9 Å cutoff was used. All bonds involving hydrogen atoms were constrained with the SHAKE (solute) or SETTLE (water) algorithm.^37,38^ The trajectory was saved every 1 ps. During simulations, a harmonic dihedral-restraint was imposed on the Gly-Pro or Ala-Pro peptide bond to keep ω_0_ = 90° and ω_0_ = 180° in the ts- and *trans*-bound systems, respectively. The force constant of the restraint potential is 200 kcal mol^–1^ rad^–2^. For each system, a single microsecond-long trajectory, rather than multiple short simulation replicas, was generated.

### Principal Component Analysis

PCA was performed on the Cartesian and dihedral coordinates of backbone atoms (N, Cα, C, and O) derived from the MD simulations. Cartesian PCA was performed using CPPTRAJ^39^ in AMBER16. Prior to dihedral PCA, Pytraj,^40^ a python package binding to the CPPTRAJ program, was used to calculate backbone dihedral φ and ψ angles for each state (free, *cis*, and ts). The angles were shifted to account for the circular nature of the data in a similar way to that implemented in the dPCA+ method^26^ and then used to generate the variance–covariance matrix characterizing the correlated internal motions. The variance–covariance matrix was then diagonalized to obtain the eigenvectors or principal components (PCs). The structural variance along each PC is given by its corresponding eigenvalue. The first two PCs, which captured the largest structural variance, were used to build the subspace where simulation trajectories were projected. To compare PCs, the RMSIP was calculated.^41^ To compare multiple systems using PCA, trajectories were concatenated prior to generating the variance–covariance matrix.

### Angular Variance and RMSF Analysis

Angular variance of the backbone and dihedral angles and residue wise RMSF were computed for all isoforms of interest. Pytraj was used to calculate the dihedral φ, ψ, and χ_1_ angles for each state. The angles were shifted in the same way as in the dihedral PCA. RMSF was calculated using CPPTRAJ in AMBER16. To better capture the dynamics during substrate binding, the angular variance or RMSF was calculated by concatenating the free and *cis* simulation frames together before calculations. Similarly, for catalysis, the *cis* and ts simulation frames were combined. The maximum variance among the dihedral angles for each residue was taken as the overall variance for that residue. For RMSF, the value was an average over all backbone heavy atoms. Results were plotted using R.^42^ To compare RMSF and angular variance between different isoforms, the Pearson correlation coefficient between the profiles of two isoforms was calculated.

### Free-Energy Calculation

The molecular mechanics Poisson–Boltzmann surface area (MM-PBSA) method^43^ was used to calculate the binding free energy between cyclophilins (CypA and CypE) and their respective substrates. Gas-phase energies were computed with the ff14SB force field. The polar term of the solvation energy was calculated by solving the linear Poisson–Boltzmann equation using the SANDER program of AMBER16. Dielectric constants of the protein interior and exterior were set to 4.0 and 80, respectively. The ionic strength was set to the default, 0.0 mM. The nonpolar solvation energy was estimated by summing up a dispersion term using a surface-based integration method^44^ and a cavity term that is linearly proportional to the molecular solvent-accessible surface area. The binding energy was calculated over every 100 frames of a simulation.

## Results and Discussion

### Cyclophilins A–E Exhibit Conserved and Divergent Intrinsic Dynamics in Individual Functional States

**Figure 2:**
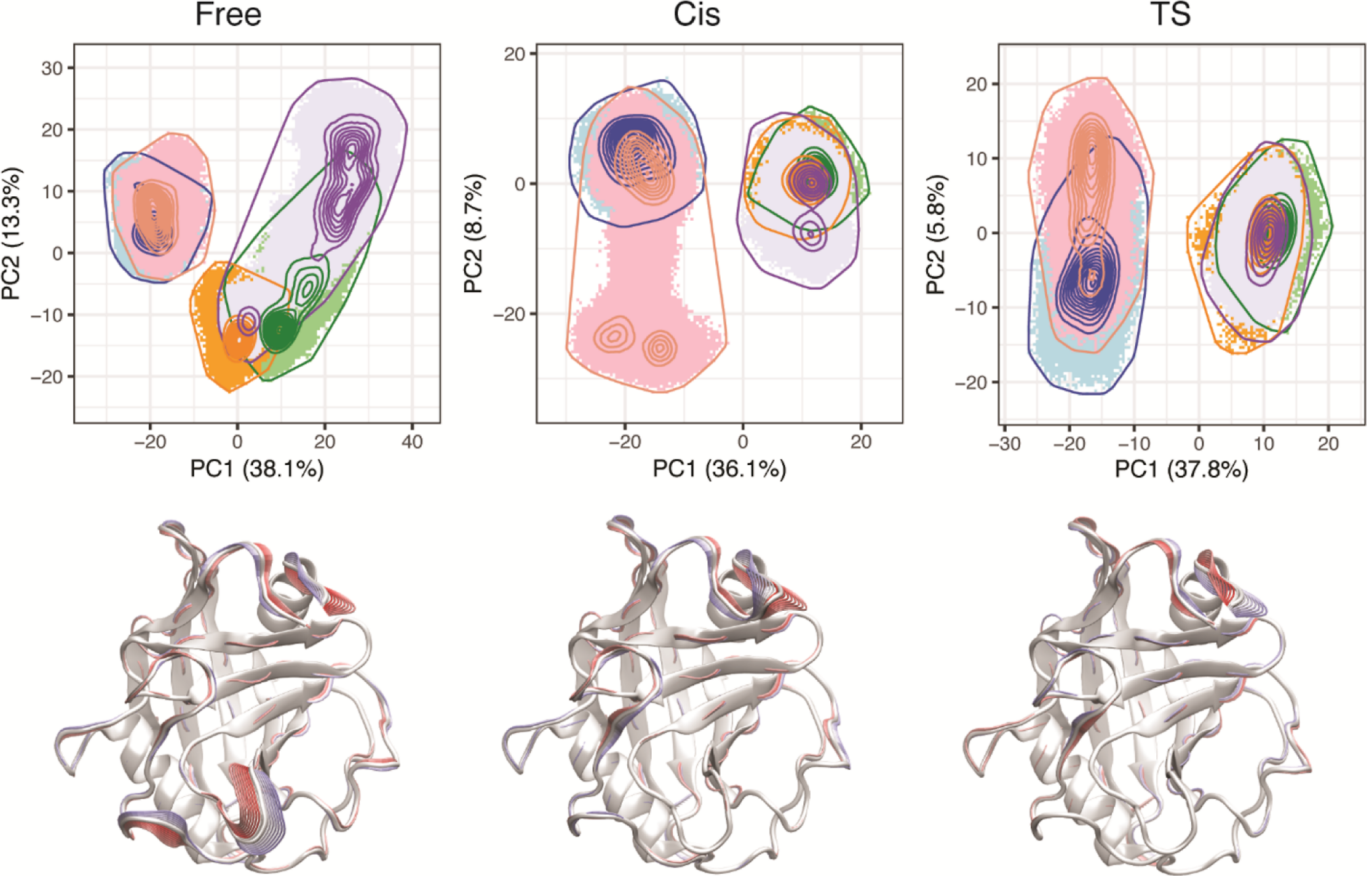
PCA comparing the dynamics of CypA (green), CypB (blue), CypC (pink), CypD (orange), and CypE (purple) in different substrate-binding conditions. (Top) Overlap of the sampled conformational space between isoforms indicates a higher similarity in dynamics. Contour lines are determined by conformational probability densities. Outlines indicate boundaries of the sampled space. The percentage of variance captured by each PC is given in parentheses in the axis label. (Bottom) Collective motion represented by each PC1. Shaded areas indicate regions of movement along PC1 (red: low; blue: high).

Multiple analyses of the MD simulation trajectories for CypA–E were performed in order to determine the similarities in dynamics among all five isoforms. Because all five isoforms are found in a variety of environments and are implicated in diverse subcellular pathways,^16,45,46^ the comparison of dynamics can provide insight into their functional conservation and divergence involving the isomerization of peptidyl-proline. PCA was performed on Cartesian backbone coordinates of each isoform in its substrate-free, substrate-*cis* (with the peptidyl-prolyl torsion angle ω = 0°), substrate-transition (ts; ω = 90°), and substrate-*trans* (ω = 180°) states. A small peptide, 11 amino acids in length, with a Gly-Pro peptide bond was used as the substrate. This peptide was derived based on the consensus motif for efficient CypA binding obtained from a phage display study.^47,48^ PCA of all five isoforms shows that CypA, CypD, and CypE share conformational space in the free, *cis*, and transition states. Similarly, CypB and CypC share conformational space between each other in the three states but are completely separate from CypA, CypD, and CypE. This indicates that the five isoforms can be divided into two subgroups: one contains CypA, CypD, and CypE, and the other contains CypB and CypC. The isoforms within each subgroup are more conserved in terms of intrinsic dynamics than those between subgroups (Figure 2). This is consistent with phylogenetic data, which indicates that CypB and CypC are more closely related to each other than CypA, CypD, and CypE, and vice versa (Figure S1). Furthermore, CypB and CypC both work in the endoplasmic reticulum,^49,50^ while CypA, CypD, and CypE mainly work in the cytosol, mitochondria, and nucleus, respectively.^18^ The different subcellular locations imply distinct substrate specificities, which might be related to the divergent dynamics of these isoforms.

To further compare the dynamics of each isoform in the free, *cis*, and transition states, the residue-wise RMSF of the backbone atoms and the angular variance of the backbone and sidechains were calculated. Both analyses demonstrate that CypA–E have highly similar patterns in the profile of fluctuations, even though magnitudes of individual residue flexibilities may diverge in the free, *cis*, and transition states (Figure S2). A more quantitative analysis looking at the correlation coefficients shows that the dynamics of the bound states (*cis* and ts) tend to be more conserved than the free state. For example, the RMSF similarity between CypC and CypE increases from 0.49 (free) to 0.67 (*cis*) and 0.69 (ts) (Figure S3). This analysis also shows that RMSF in all three states of CypA shares greater similarity with CypD and CypE than CypB or CypC, again highlighting that CypB and CypC are more closely related to each other than CypA, CypD, and CypE, and vice versa.

### Cyclophilins A–E Share Conserved Dynamics during Substrate Binding and Catalysis

**Figure 3:**
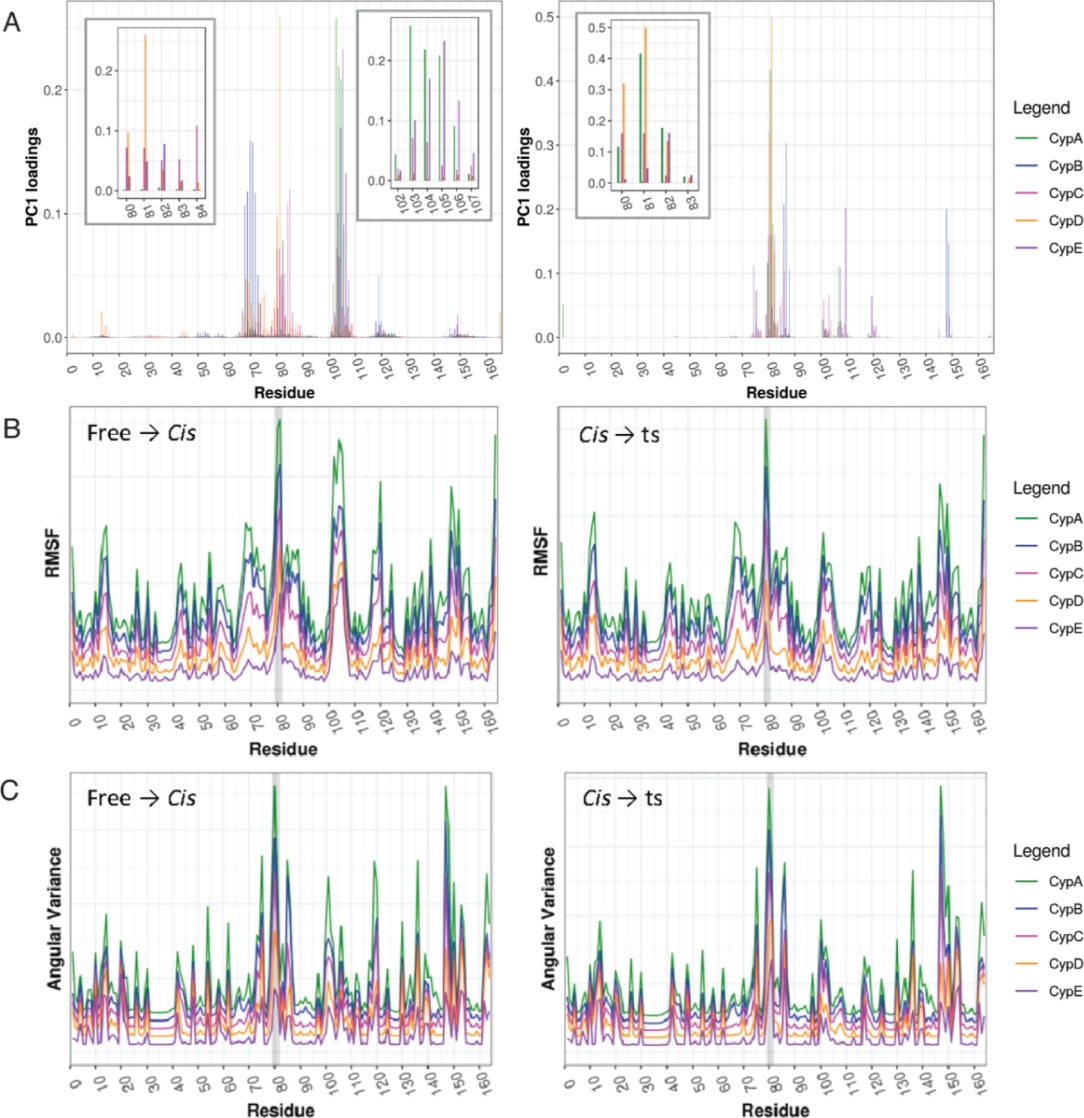
Cyclophilins A–E share conserved dynamics. (A) Contribution of each residue to Cartesian (right) and dihedral (left) PC1. Inset figures show regions of conserved high contribution. (B) RMSF of each residue from free to *cis* (left) and *cis* to ts (right). Within each protein, RMSF values fluctuate in the unit of Å. (C) Angular variance of each residue from free to *cis* (left) and *cis* to ts (right). The lines for each isoform are shifted to avoid overlaps in (B,C); thus, the ordinate scales are omitted, and the relative trends of the lines are compared. The gatekeeper 2 region is highlighted by a light-gray background.

To gain further insight into the conserved dynamics of CypA–E and in an effort to identify key residues involved in the dynamics that may be related to the catalytic activity, PCA was performed over a mixture of the substrate-free, *cis*, and transition-state conformational ensembles for each isoform. For each isoform, PCA was performed on both the Cartesian and dihedral coordinates of the backbone, and the contribution of each residue to the first principal component (PC1) was analyzed. Through this, we were able to identify the residues that contribute most to the dominant protein motion. The result shows that for most isoforms, the highest contribution to the dominant dynamics comes from the gatekeeper 2 region (residues 80–83, with respect to the CypA sequence). In the dihedral PCA, the residues that contribute most to PC1 in CypB slightly shift toward the C-terminus from gatekeeper 2 (around residue 86). In the Cartesian PCA, however, those PC1-dominant residues in CypB slightly shift toward the N-terminus from gatekeeper 2 (around residue 71, close to gatekeeper 1), while those in CypA and CypE shift to a loop closer to the active site (residues 102–107, next to gatekeeper 3) (Figure 3A).

To confirm the high contribution of the gatekeeper 2 region to the function-related dynamics of cyclophilins, analyses of the residue-wise RMSF of the backbone atoms and the angular variance of the backbone and sidechains across conformational ensembles representing distinct substrate-binding conditions were performed. Both analyses demonstrate that CypA–E have very similar profiles of residue flexibility, indicating highly conserved dynamics across the isoforms. Notably, the gatekeeper 2 region is very flexible in all isoforms, and in almost all cases (except for RMSF during substrate binding in CypE, where the flexibility of the loop 102–107 is slightly higher), this region exhibits the highest flexibility (Figure 3B,C). This is slightly different from the PCA, where a shift of PC1-dominant residues away from gatekeeper 2 is observed in CypA, CypB, and CypE. Nonetheless, both analyses identify the gatekeeper 2 region as making conserved dynamical contributions to both substrate binding (free → cis) and catalysis (cis → ts), suggesting the importance of this region to enzyme function.

A more quantitative measurement of similarities is provided by the Pearson correlation coefficient between the profiles of flexibility of two isoforms (Figure S4). On average, the catalytic step (cis → ts) has a slightly higher correlation coefficient (RMSF: 0.73) than the substrate binding (free → *cis*) (0.66), suggesting that catalysis is more dynamically highly conserved. This is consistent with our previous MD study comparing CypA, CypD, and CypE using residue–residue contacts and the study combining MD and quasi-harmonic analysis to examine multiple families of enzymes including cyclophilins.^7,11^ Both previous studies support the notion that the dynamics coupled to critical chemical steps, which are likely inherited from a common ancestor of the enzyme family during evolution, are highly conserved.

### Highly Conserved Glycine Is Responsible for the High Flexibility of the Gatekeeper 2 Region

The sequence alignment of all 17 human cyclophilins shows multiple highly conserved glycines across catalytic isoforms.^15^ One of these glycines, Gly80, located in the gatekeeper 2 region is present in all catalytic isoforms except PPIH (Figure S5). We hypothesized that this glycine was responsible for the high flexibility of the gatekeeper 2 loop region, given that glycine is the smallest amino acid. To test this hypothesis, we constructed CypA and CypE mutants, where Gly80 was replaced with an alanine (G80A). The additional sidechain alanine atoms increase the bulkiness, thus decreasing flexibility. CypA was chosen for mutation due to the available wealth of additional experimental data, which would help validate our findings. CypE was chosen as it appeared to have the largest overall flexibility during substrate-binding (backbone average RMSF: 0.871 Å) and catalytic processes (0.847 Å); therefore, any changes in the mutant should be more obvious.

**Figure 4:**
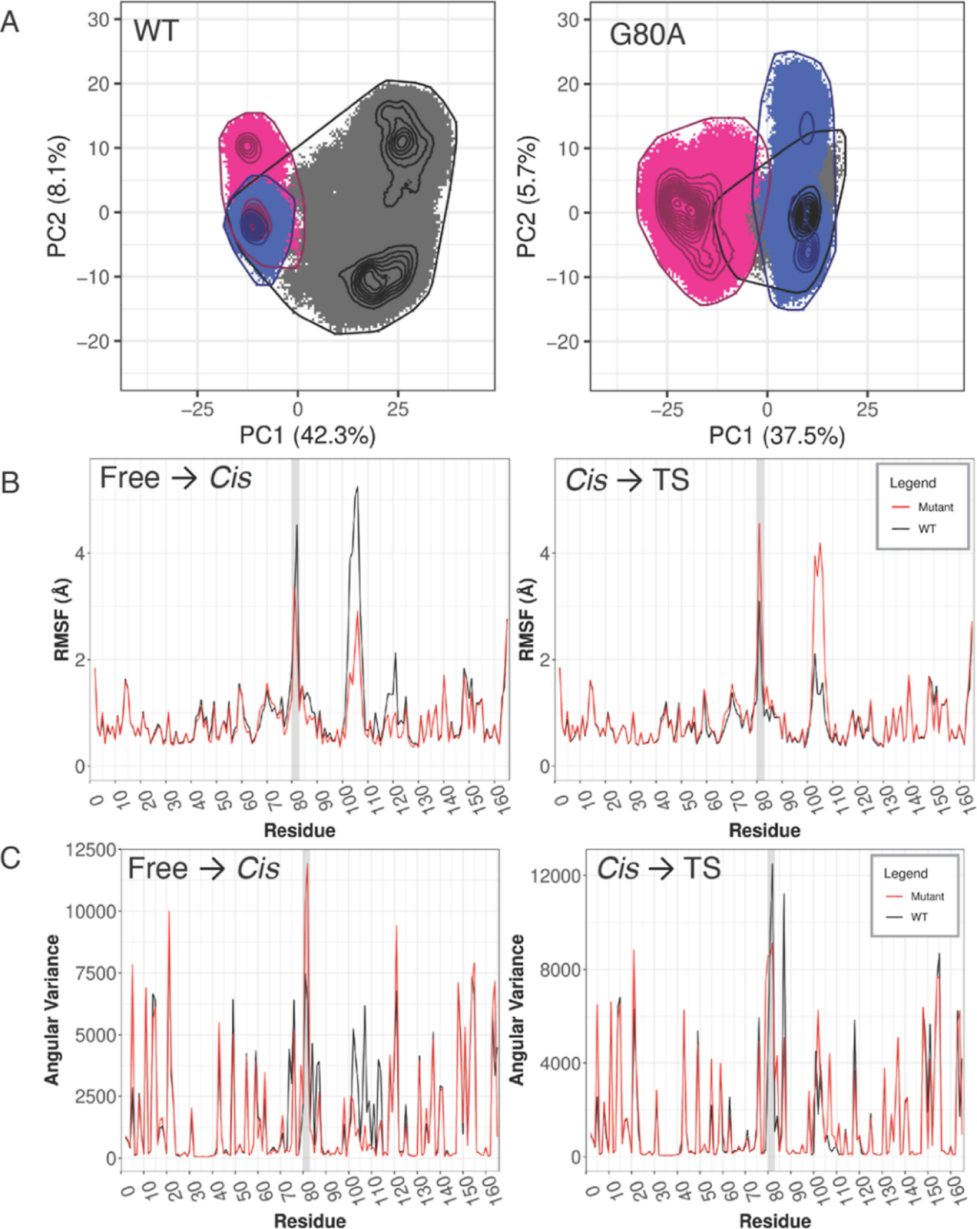
Mutation G80A in CypE leads to increased rigidity during substrate binding but increased flexibility during catalysis. (A) Cartesian PCA of WT and mutant CypE comparing free (black), *cis* (pink), and ts (blue). Root mean square inner product (RMSIP) of top 10 PCs between the WT and the mutant is 0.7; a RMSIP close to one indicates high similarity between PC spaces. (B) RMSF and (C) angular variance of WT and mutant CypE. The gatekeeper 2 region is highlighted by a light gray background.

Additional 2 μs MD simulations were performed for the mutants under substrate-free, *cis*, and transition states. PCA was used to analyze global changes in dynamics during substrate-binding and catalytic processes for the WT and the mutant. PCA of CypE combining the substrate-free, *cis*, and transition states shows that the conformational space sampled by the free state is smaller in the mutant, as compared to the WT (Figure 4A). This indicates an increased rigidity of the protein in its apo form, consistent with our hypothesis that Gly80 is responsible for the large flexibility of the gatekeeper 2 region. Interestingly, the conformational space sampled by the *cis* state in the mutant is almost entirely separated from that of the transition state. In contrast, in the WT, the conformational space sampled by the two states overlaps greatly, with one of the two wells of the *cis* state overlapping almost completely with the well of the transition state (Figure 4A). This suggests that mutated CypE exhibits increased flexibility during catalysis. This increase in flexibility may be forced due to additional steric effects in the substrate–enzyme interaction that would arise as a result of the additional side-chain atoms in alanine.

PCA provides information about the global, collective backbone conformational dynamics. To gain further insight into the local effects of the G80A mutation in CypE, we also looked at the backbone RMSF and angular variance of the backbone and side chains. Consistent with PCA, RMSF is overall lower for the mutant during substrate binding and higher for the catalysis (Figure 4B,C). Notably, the mutant has a lower RMSF in the gatekeeper 2 region but an increased angular variance during substrate binding. The enhanced flexibility of internal angular degrees of freedom may compensate for the loss of entropy due to the reduced RMSF. Similarly, during catalysis, the RMSF in the gatekeeper 2 region is increased in the G80A mutant, while the angular variance is lower, possibly to compensate for the RMSF change. The G80A CypE mutant also shows significant RMSF and angular variance changes in another region downstream of the mutation (residues 101–111). This region contains the loop comprising the gatekeeper 3 region.^15^ In this region, changes of both RMSF and angular variance follow the same trend of the RMSF changes in gatekeeper 2, that is, decrease during substrate binding and increase during catalysis. In summary, these data combined with the results of PCA suggest that the G80A CypE mutant exhibits overall increased rigidity during substrate binding and increased flexibility during catalysis, and the dynamical changes mainly occur in the gatekeeper 2 and gatekeeper 3 regions.

**Figure 5:**
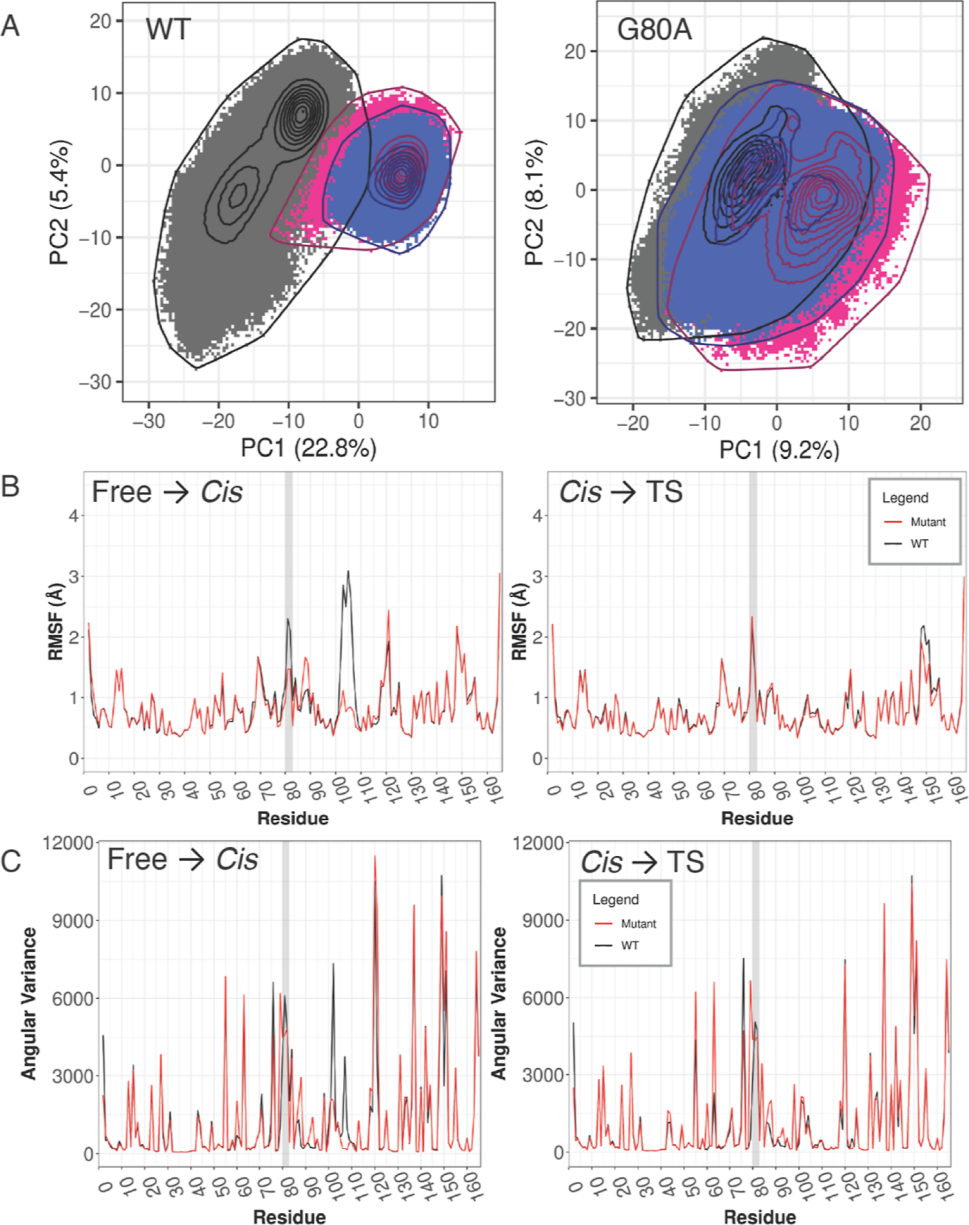
Mutation G80A in CypA shows dynamical changes similar to CypE. (A) Cartesian PCA of WT and mutant CypA comparing free (black), *cis* (pink), and ts (blue). The RMSIP of top 10 PCs between WT and mutant is 0.16. (B) RMSF and (C) angular variance of WT and mutant CypA. The gatekeeper 2 region is highlighted by a light gray background.

To verify that these results are not specific to a single isoform of human cyclophilin, the effects of mutation G80A on CypA were also analyzed. Consistent with the previous results, PCA of CypA combining the substrate-free, *cis*, and transition states shows that the conformational space sampled by the free state in the mutant has greater overlap with that of the *cis* state, as compared to the WT (Figure 5A), indicating increased rigidity during substrate binding. On the other hand, the conformational space sampled by the *cis* state in the mutant shows greater separation from that of the transition state, as compared to the WT (Figure 5A), indicating increased flexibility during catalysis. However, the magnitude of changes in CypA is smaller than that in CypE. For example, the intrinsic flexibility of the free state is largely reduced in CypE, but it does not change so much in CypA. Also, where mutant CypE shows almost no overlap between the *cis* and the transition state, mutant CypA still maintains overlap; an observation of the contour lines, which indicate the most highly sampled conformations, indicates that there is merely a decrease in overlap between the highly sampled conformations. The difference in severity of the effect of the G80A mutation on dynamics between CypA and CypE is to be expected: WT CypE has much higher flexibility in the gatekeeper 2 region than WT CypA, indicating greater room for the G80A mutation to modulate dynamics, thus producing more pronounced mutational effects in CypE.

RMSF and angular variance were then analyzed to obtain a better understanding of the local effects of the G80A mutation in CypA. Both RMSF and angular variance are overall lower for the mutant during substrate binding, whereas they increase slightly during catalysis (Figure 5B,C). Similar to CypE, the CypA G80A mutant has a lower RMSF in both gatekeeper 2 and gatekeeper 3 regions during substrate binding compared to the WT; however, even though angular variance decreases in the gatekeeper 3 region as in CypE, it also slightly decreases in gatekeeper 2 as opposed to that in CypE. Interestingly, although the gatekeeper 2 region exhibits slightly increased rigidity during catalysis, multiple other sites, including gatekeeper 3, show increased flexibility upon the G80A mutation, which both supports the PCA result and is similar to CypE. Together, these data demonstrate that dynamic changes in CypA as a result of the G80A mutation are similar to, although less pronounced than, those exhibited by CypE, suggesting that the effects of the mutation on dynamics are not specific to just one cyclophilin isoform.

### G80A Mutation Reduces the Catalytic Efficiency of CypA and CypE

To further understand the effects of the G80A mutation on enzyme catalysis, free-energy distributions of isomerization for both WT and mutant cyclophilins were analyzed. We emphasize that although understanding enzyme catalysis generally requires quantum mechanics (QM) calculations, PPIase-catalyzed prolyl isomerization represents a special case where classical molecular mechanics (MM) is sufficient. It has been generally accepted that PPIase catalysis does not involve any covalent bond formation or breakage.^51^ The polarization effect during isomerization is also small according to our previous QM calculations,^52^ supporting the use of a fixed-charge force field; such an effect was suspected due to the transition from a planar (*cis* or *trans*) to a pyramidal (transition state) geometry of the amide nitrogen. Moreover, previous computational studies using classical methods have generated results consistent with experiments, including reproducing the 6–9 kcal/mol activation energy reduction (equivalent to 4–6 orders of magnitude of rate acceleration) by CypA.^52−54^

Instead of directly computing the potential of mean force along the reaction, we calculated the binding free energy between the substrate and the enzyme in the *cis*, transition, and *trans* states using the MM-PBSA method.^43^ Here, we follow the well-established transition-state stabilization mechanism of enzyme catalysis. If the substrate in the transition state has a stronger binding affinity (lower binding free energy, Δ*G*) than ground states, the free-energy barrier for the reaction to proceed is reduced, i.e., the isomerization of the substrate can be catalyzed by the enzyme; otherwise, catalysis does not occur. The larger the binding free energy difference is, the more the energy barrier is reduced. Hence, by examining the relative binding free energies of the transition and ground states, we can obtain information about how the mutation affects catalysis. More detailed discussion about the approach focusing on PPIases is provided elsewhere.^55^ For these analyses, additional 2 μs simulations were performed for WT and mutants CypA and CypE with the substrate in the *trans* conformation.

**Figure 6:**
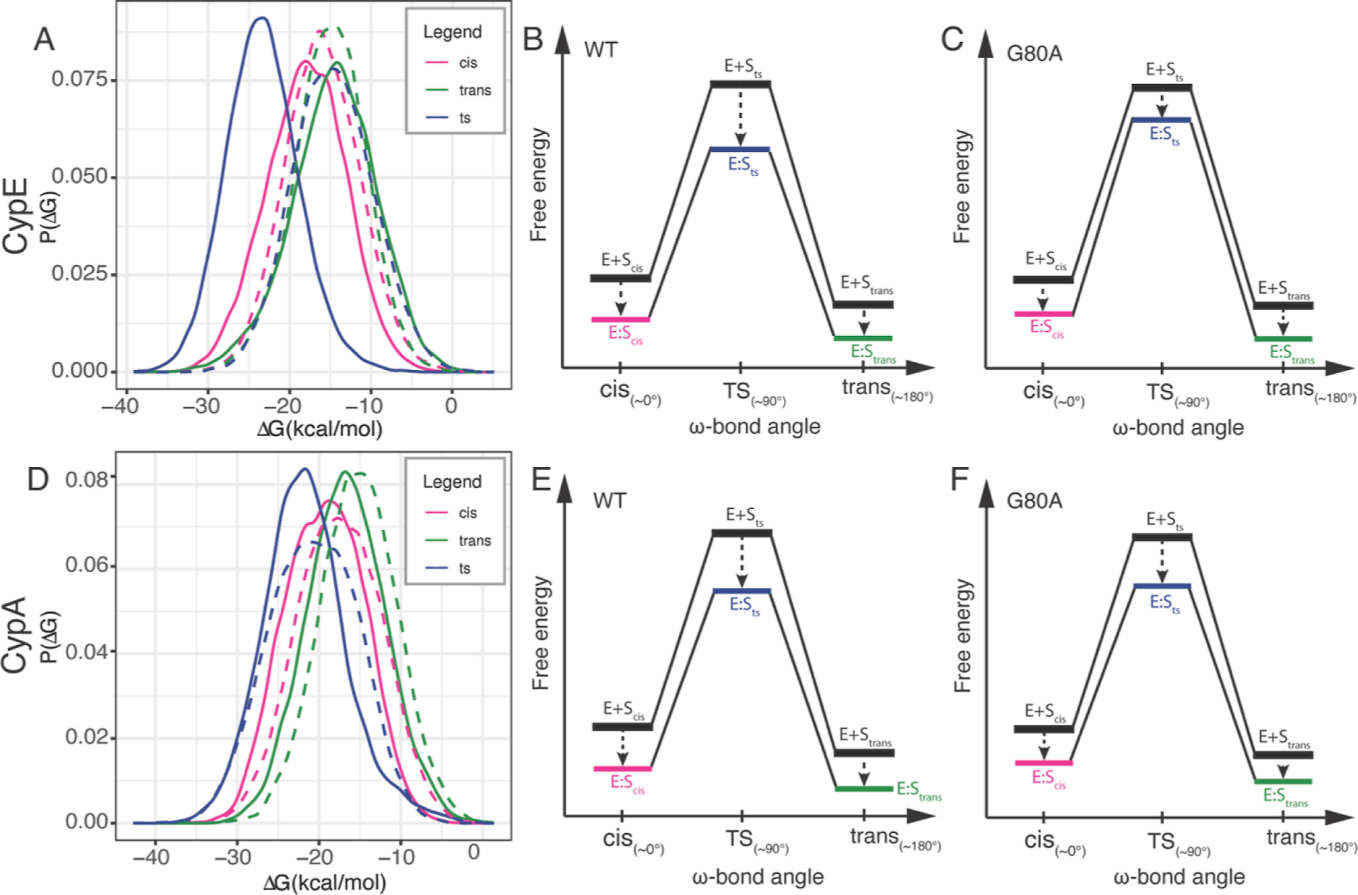
Binding free energy analysis of WT and mutants CypE and CypA. (A) Binding free energy distributions between CypE and the substrate in different conformations. Solid lines indicate WT, and dashed lines indicate mutants. Schematic free-energy diagrams derived based on the calculated binding energies for (B) WT and (C) mutant CypE. E + S and E/S represent the unbound and bound states, respectively. (D–F) Results for WT and mutant CypA.

Results for CypE show that the mean binding free energy of the WT in the transition state (−23.33 kcal/mol) is lower than that in the *cis* (−17.98 kcal/mol) and *trans* (−14.59 kcal/mol) conformations (Figure 6A). This indicates that the enzyme can catalyze the isomerization from both ground states with similar efficiency (Figure 6B). The mean binding free energy of the CypE G80A mutant in the transition state (−14.69 kcal/mol) is similar to that of the *cis* state (−15.95 kcal/mol) and *trans* state (−15.10 kcal/mol; Figure 6A). This energetic similarity indicates an unaltered energy barrier for the isomerization within the enzyme (and hence no catalytic activity for the mutant) (Figure 6C).

The catalytic efficiency is also reduced by the G80A mutation in CypA, although the magnitude of effect is smaller than that in CypE. The mean binding free energy of the WT in the transition state (−21.67 kcal/mol) is lower than that of the *cis* (−19.31 kcal/mol) and *trans* (−16.68 kcal/mol) states (Figure 6D), again indicating that the enzyme can catalyze the isomerization from both ground states with similar efficiency (Figure 6E). In the CypA G80A mutant, we see a similar energy barrier reduction; the mean binding free energy in the transition state (−20.73 kcal/mol) is lower than that of the *cis* (−17.53 kcal/mol) and *trans* (−15.18 kcal/mol) states (Figure 6D). The relative differences of mean binding free energy between the *cis*, ts, and *trans* states remain the same across the mutant and WT. However, the mutant has a slightly lower binding affinity for both ground states, indicating a reduced catalytic efficiency compared to the WT CypA. The small decrease in efficiency here is likely because the Gly-Pro peptide is not a preferred substrate of CypA. Tests on another peptide (Ala-Pro) that can be more efficiently catalyzed by CypA indeed show a more pronounced difference in catalytic activity, similar to that observed for CypE (see below).

### Similar Dynamic and Catalytic Changes by the G80A Mutation Are Observed Using a Different Substrate in CypA.

To test the dependence of the results on the identity of the substrate, the effects of mutation G80A on CypA were analyzed using a different peptide containing an Ala-Pro peptide bond placed in the active site. Additional 2 μs simulations were performed for CypA with the peptide in the *cis*, ts, and *trans* states.

**Figure 7:**
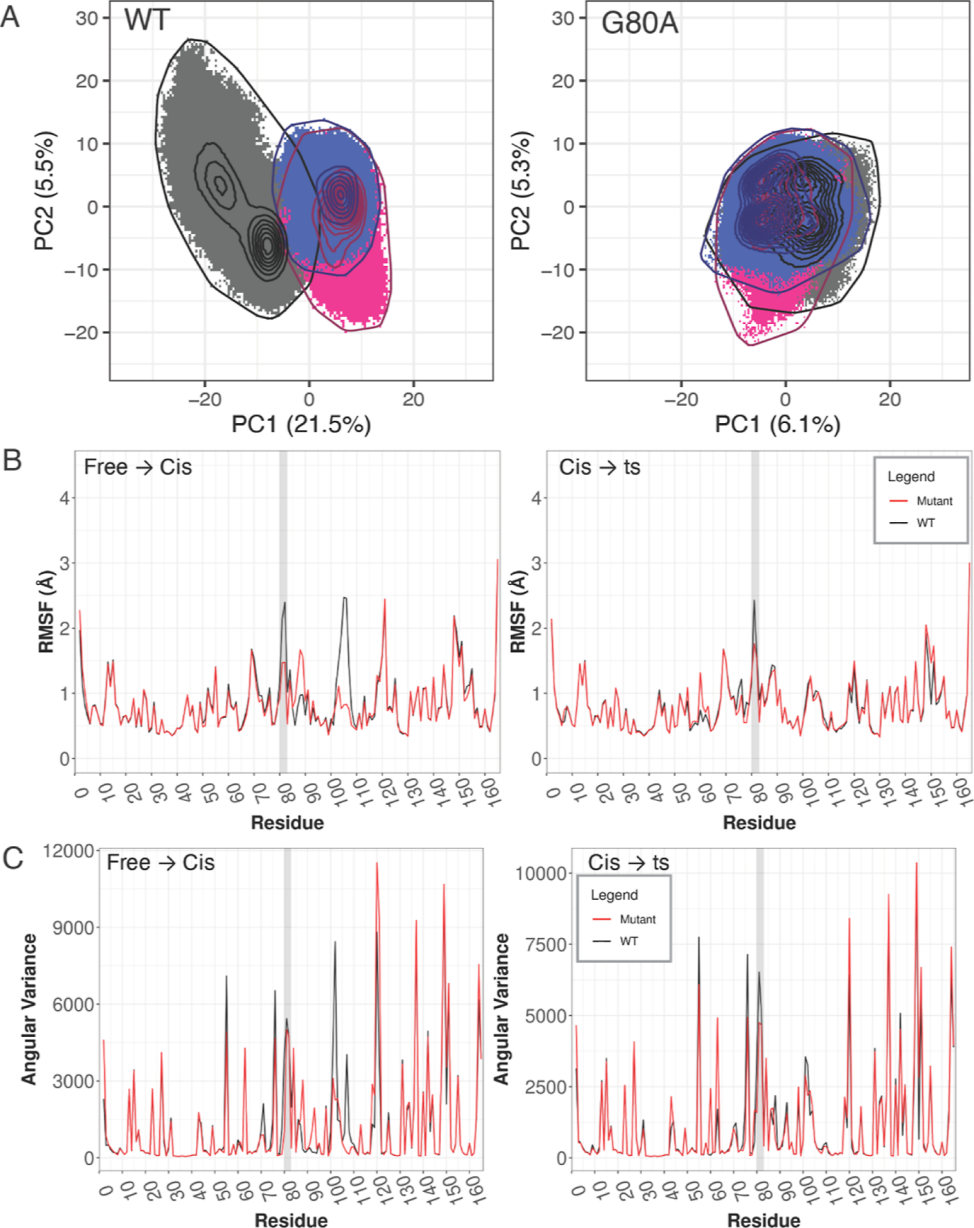
Dynamical changes by the mutation G80A in CypA using the Ala-Pro peptide. (A) Cartesian PCA of WT and mutant CypA comparing free (black), *cis* (pink), and ts (blue). RMSIP of top 10 PCs is 0.26. (B) RMSF and (C) angular variance of WT and mutant CypA. The gatekeeper 2 region is highlighted by a light gray background.

The PCA of CypA combining the substrate-free, *cis*, and transition states shows that the conformational space sampled by the mutant in the free state has largely increased overlap with the *cis* state as compared to the WT (Figure 7A). This indicates that, as with the Gly-Pro peptide, the CypA G80A mutant exhibits increased rigidity during substrate binding. It appears that the mutant maintains similar overlap of the *cis* and transition states with respect to the overall sampled space as compared to the WT. However, observation of the contour lines does indicate the formation of two different wells in the transition state of the mutant. One of these wells does not overlap with the well of the *cis* state at all. This could indicate some increase in flexibility during catalysis. This is similar to what is observed with the Gly-Pro peptide, although the magnitude of change is much smaller here.

RMSF and angular variance are both overall lower during substrate binding for the mutant as compared to the WT. The gatekeeper 2 and 3 regions display the most drastic decrease in both analyses (Figure 7B,C). Similar to CypA with the Gly-Pro peptide, the CypA G80A mutant with the Ala-Pro peptide has a lower RMSF and angular variance in the gatekeeper 2 region during catalysis, but a slight increase in the two in various other regions, including the gatekeeper 3 region. Together, these data demonstrate that dynamic changes in CypA as a result of the G80A mutation with the Ala-Pro peptide are similar to those exhibited with the Gly-Pro peptide, suggesting that the mutational effects on dynamics may be independent of the substrate sequence.

**Figure 8:**
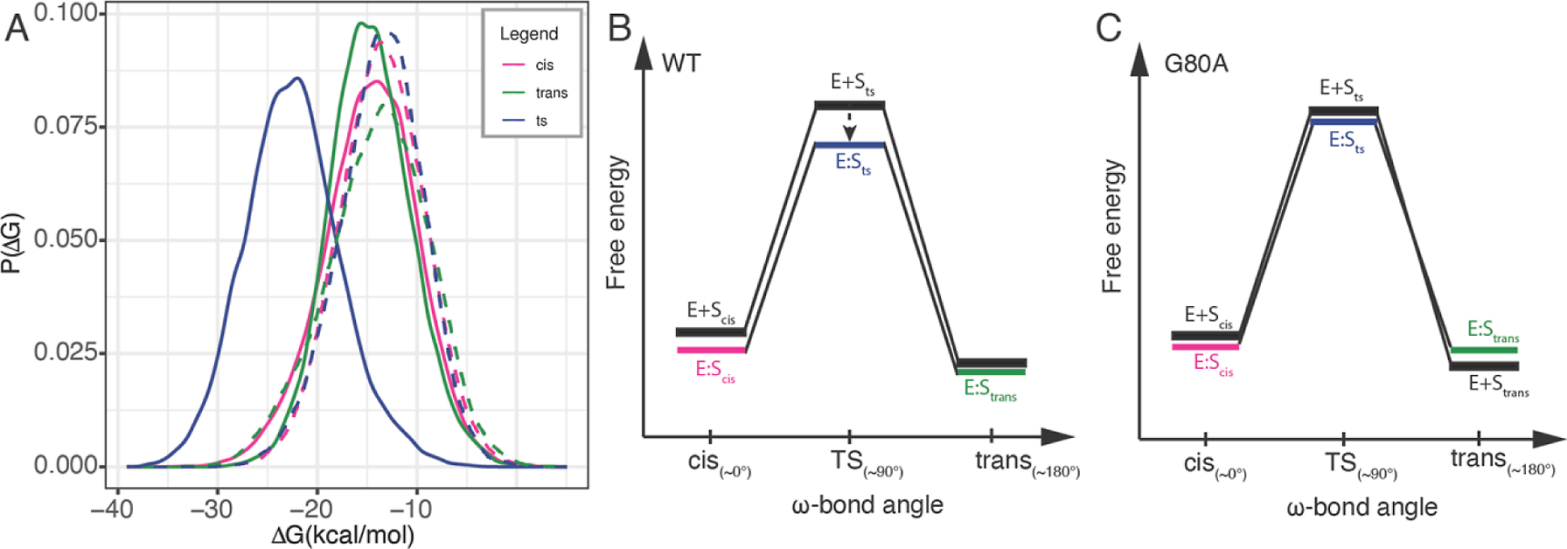
Binding free energy analysis for WT and mutant CypA bound with the Ala-Pro peptide. (A) Binding free-energy distributions. Solid lines indicate WT, and dashed lines indicate mutants. Schematic free-energy diagrams derived based on the calculated binding energies for the (B) WT and (C) mutant. E + S and E/S represent the unbound and bound states, respectively.

The mutational effects on catalysis were then examined through the analysis of binding free energies calculated by MM-PBSA. The mean binding free energy of the WT in the transition state (−22.47 kcal/mol) is much lower than that of the *cis* (−14.70 kal/mol) and *trans* (−14.65 kcal/mol) states (Figure 8A). This indicates that the WT can catalyze the transition from *cis* and *trans* states with similar efficiency (Figure 8B). In the mutant, we see similar mean binding free energies in the *cis* (−13.53 kcal/mol), ts (−13.61 kcal/mol), and *trans* (−13.95 kcal/mol) states (Figure 8A). This suggests that the G80A mutation leads to a loss in catalytic ability of the enzyme for the Ala-Pro peptide (Figure 8C). This is similar to the results observed with the Gly-Pro peptide in CypE, where the G80A mutation also causes a dramatic decrease in catalytic efficiency. In CypA, the difference is less significant when using the Gly-Pro peptide, possibly because the Gly-Pro peptide bond is not a preferred substrate of CypA. It has been shown that the Ala-Pro peptide bond in the active site is more preferred by CypA;^56^ with Ala-Pro peptide, we indeed see a more significant loss of catalytic activity in the G80A mutant.

Another possible reason to explain the small decrease in catalytic efficiency observed with the G80A mutant and Gly-Pro peptide is the neglection of configurational entropy in our binding free-energy calculations. Upon binding of a substrate to an enzyme, there is an entropic cost due to the loss of external and internal degrees of freedom of the system. It has been controversial whether including this cost will benefit or bring more errors in the binding free-energy calculations,^4,35^ and hence, many studies have neglected this cost assuming that the configurational entropic change upon binding is small. However, we cannot exclude the possibility that the configurational entropic effect is significant in the case of Gly-Pro binding to CypA. a careful examination of the entropic effect (using, for example, the cumulant expansion method)^57,58^ or calculation of the free-energy profile using more rigorous methods such as umbrella sampling^59^ may help uncover any small changes in catalytic efficiency. In summary, our data indicate that although the magnitude of change varies between CypA and CypE or when using different peptide substrates, the effect of the G80A mutation on catalytic ability is in general isoform and substrate-sequence independent.

## Conclusions

In this study, we identify key dynamic residues in the catalytic activity of human cyclophilins using a dynamical evolution analysis. The closely related homologues CypA–E are highly conserved in structure but show both conserved and divergent intrinsic dynamics. During functional processes, including substrate binding and catalysis, the dynamics, described by residue wise flexibilities, of the five cyclophilin isoforms are highly conserved. The region of the highest flexibility is found in the gatekeeper 2 region, which allosterically regulates access to the active site. This region also contains a highly conserved glycine (Gly80), which is hypothesized to allow for greater flexibility of the region. The mutation of Gly80 to alanine in CypE leads to an overall increased rigidity in substrate binding but increased flexibility in catalysis. These dynamic changes occur both in the mutated region (gatekeeper 2) as well as another loop downstream of the mutation site (gatekeeper 3 region). The same mutation in CypA produces similar changes, although the magnitude of changes is less pronounced in CypA than it is in CypE. Similar mutation-induced dynamic changes are observed with both the Gly-Pro peptide and the Ala-Pro peptide in CypA.

The effect of the G80A mutation on the catalytic activity is more profound and likely isoform and substrate-sequence independent. In both CypE and CypA, when using two different peptide substrates, we observe a decrease in catalytic efficiency upon the mutation. The magnitude of difference is smaller when using the Gly-Pro peptide in CypA, indicating a potential sequence dependency on the detailed mutational effect. Such a slight discrepancy may also be due to a limitation of our method, i.e., the ignorance of the configurational entropy in the binding free-energy calculations. Future work using a more accurate binding free-energy method may help clarify this uncertainty. So far, our results suggest that the mutational effect is in general independent of the substrate sequence, at least in CypA.

Another approach to assessing catalytic activity is to examine the so-called “near attack conformation (NAC)” generated during simulations. The approach can be convenient when the catalysis involves, for example, covalent bond formation and breakage, but a complex quantum mechanical modeling is to be avoided.^60^ As PPIase-catalyzed prolyl isomerization can be fully described by classical methods, the current approach examining binding free energies and using the transition-state stabilization theory is more straightforward. Note that the population of NACs in CypA has been examined previously, but controversial conclusions about its relevance to enzyme catalysis have been obtained.^61,62^

The 17 human cyclophilin isoforms encompass a wide range of sequence identities (32–75%) and functions, making them good model systems to determine how sequence variations modulate enzyme functions. In particular, the protein family contains both catalytic and noncatalytic isoforms, even though all the isoforms are structurally similar and can bind protein substrates in the well-conserved pocket. An important extension of our work will be to compare the entire cyclophilin family: Whereas comparing across catalytic isoforms may help to understand the fine tuning of the enzyme function through evolution, comparing between catalytic and noncatalytic isoforms may help to gain insights into modifications of dynamics that are disallowed if the catalytic activity is to be maintained. In both types of comparisons, in addition to conserved dynamics, divergent dynamics across isoforms will also be a focus, which may underlie functional specificities in synergy with sequence variations.

## Data Availability

Analysis scripts, input files, and trajectories of MD simulations are available upon reasonable request to Dr. Donald Hamelberg at dhamelberg@gsu.edu.

## Supporting Information Available

Phylogenetic tree detailing the evolutionary relationship between all 17 isoforms of human cyclophilin; RMSF and angular variance of the free, *cis*, and transition states of cyclophilins A–E; Pearson correlation coefficients of RMSF and angular variance for single states; Pearson correlation coefficients of RMSF and angular variance between cyclophilin isoforms over substrate binding and catalysis; and a highly conserved glycine found in the gatekeeper 2 region (PDF)
