# Supplementary material for: Conserved Conformational Dynamics Reveal a Key Dynamic Residue in the Gatekeeper Loop of Human Cyclophilins
Source: J Phys Chem B. 2023 Mar 29;127(14):3139–50. doi: 10.1021/acs.jpcb.2c08650 (PMC10108351; doi:10.1021/acs.jpcb.2c08650)
Supplement: Supplementary file 1 — jp2c08650_si_001.pdf [file jp2c08650_si_001.pdf]

Supporting Information for

Conserved Conformational Dynamics Reveal a Key Dynamic Residue in the Gatekeeper Loop of Human Cyclophilins

Furyal Ahmed[1,2], Xin-Qiu Yao[1], Donald Hamelberg*[1]

[1]Department of Chemistry, Georgia State University, Atlanta, Georgia 30302-3965, USA

[2]Agnes Scott College, Decatur, Georgia 30030, USA

*Corresponding Author. Tel.: (404) 413-5564; E-mail: dhamelberg@gsu.edu

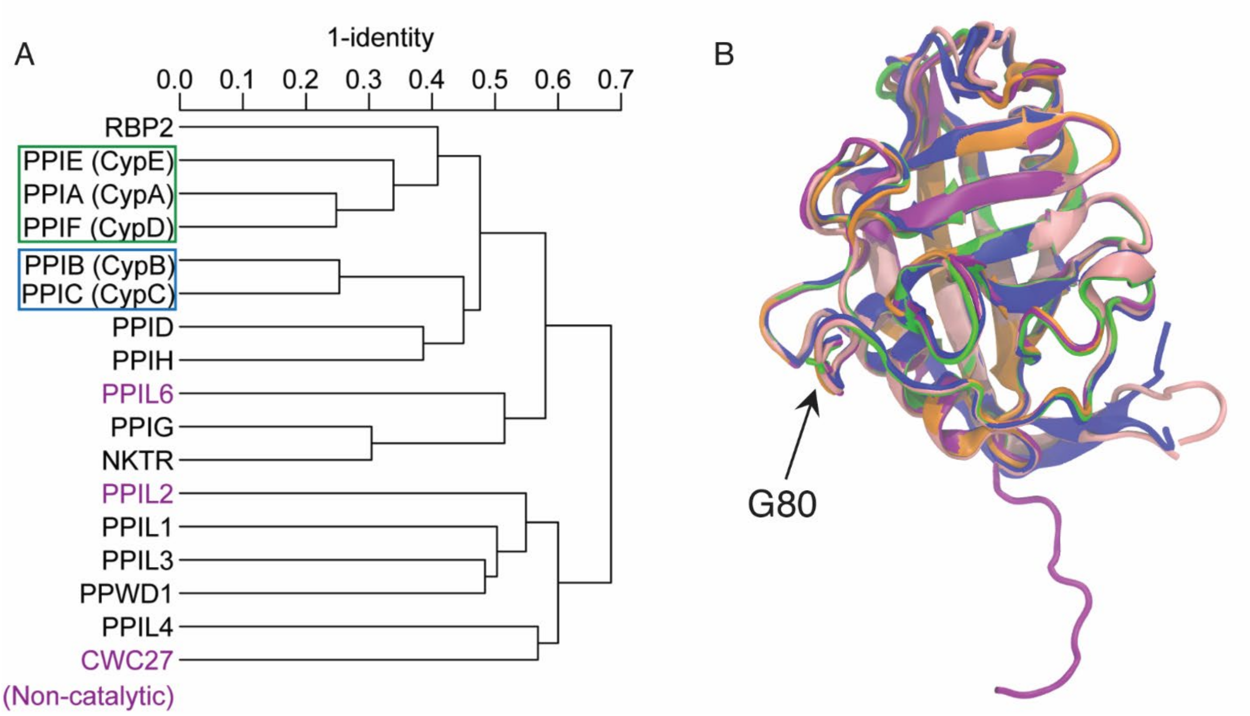

Figure S1. (A) Phylogenetic tree detailing the evolutionary relationship between all 17 isoforms of human cyclophilin. (B) Superimposed structures of CypA (green), CypB (blue), CypC (pink), CypD (orange), and CypE (purple). The G80 (or equivalent) residues are shown as sticks color coded the same as the backbone.

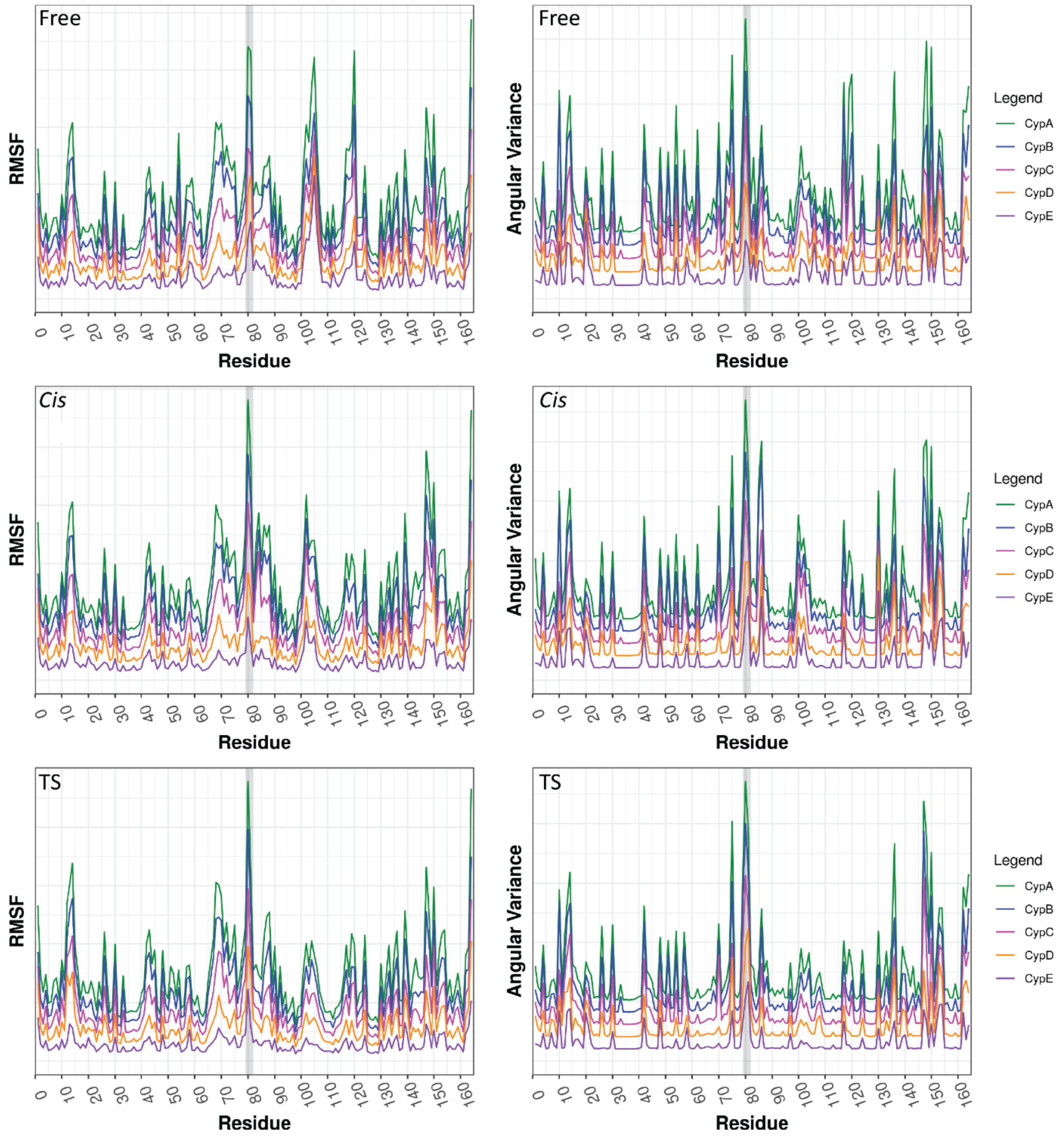

Figure S2. RMSF and angular variance of the free, *cis*, and transition state of cyclophilins A-E.

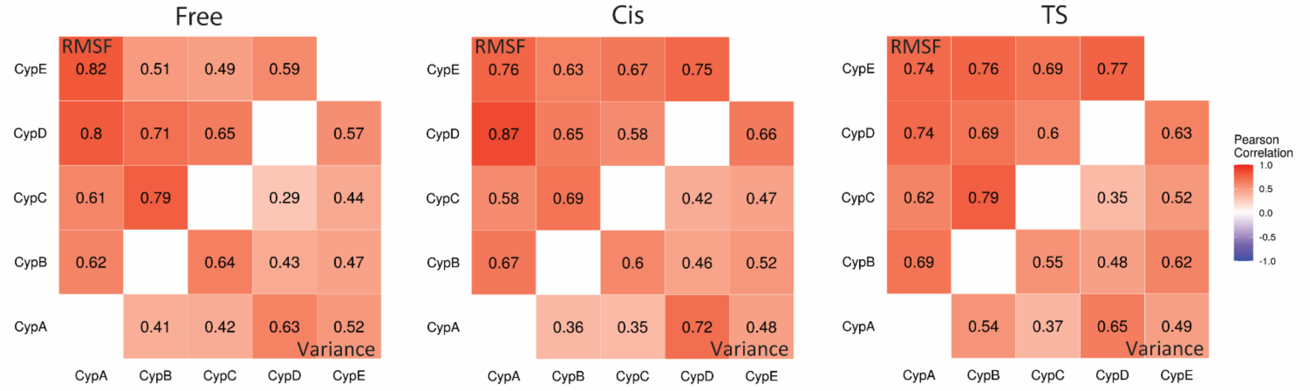

Figure S3. Pearson correlation coefficients of RMSF and angular variance for single states. Lower triangular shows data for angular variance. Upper triangular shows data for RMSF.

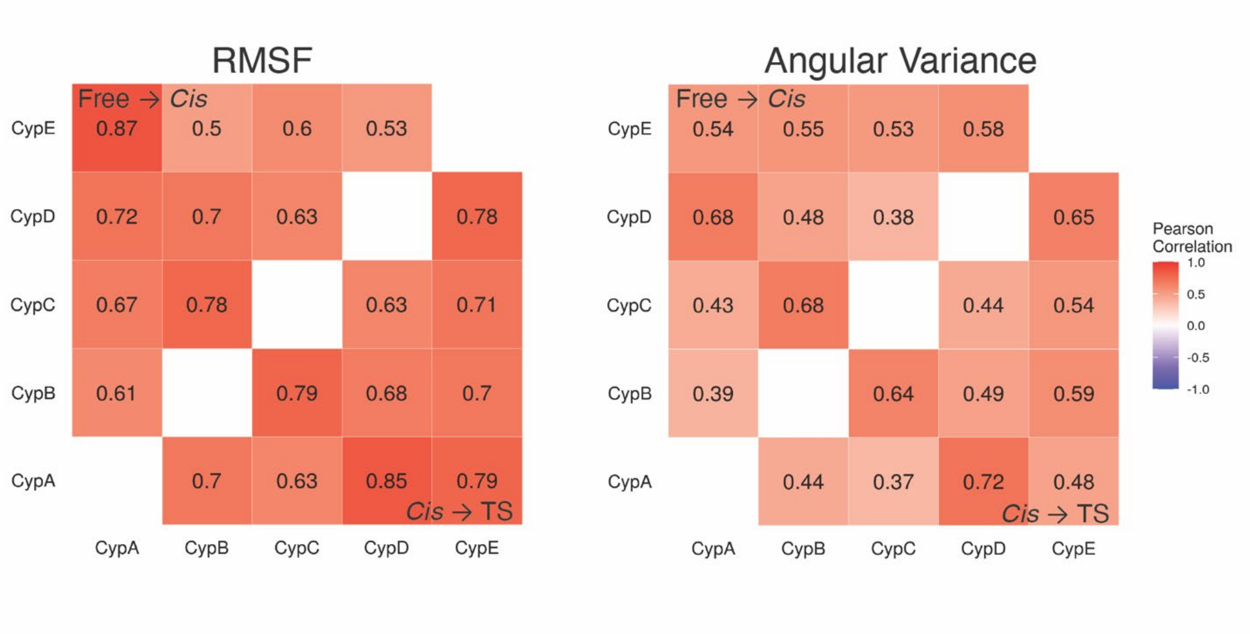

Figure S4. Pearson correlation coefficients of RMSF and angular variance between cyclophilin isoforms. A coefficient closer to one indicates higher similarity. Upper triangular shows data for substrate binding (free → *cis*). Lower triangular shows data for catalysis (*cis* → ts).

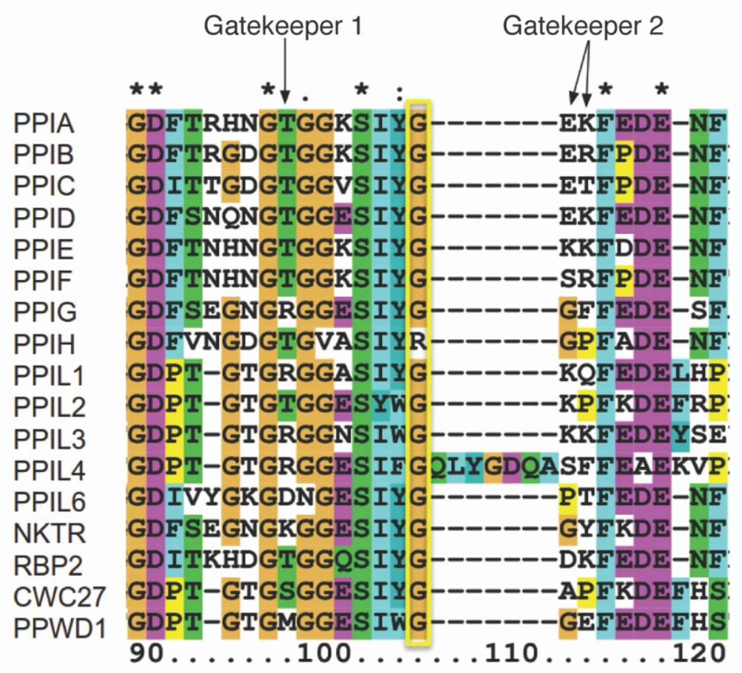

Figure S5. A highly conserved glycine is found in the gatekeeper 2 region. Sequence alignment of all 17 human isoforms of cyclophilin showing gatekeeper regions. Highly conserved glycine in gatekeeper 2 region is outlined in yellow. Alignment is generated using ClustalX.[1]

References

(1) Larkin, M. A.; Blackshields, G.; Brown, N. P.; Chenna, R.; McGettigan, P. A.; McWilliam, H.; Valentin, F.; Wallace, I. M.; Wilm, A.; Lopez, R.; Thompson, J. D.; Gibson, T. J.; Higgins, D. G. Clustal W and Clustal X Version 2.0. *Bioinforma. Oxf. Engl.* **2007**, *23* (21), 2947–2948. https://doi.org/10.1093/bioinformatics/btm404.
